# Barriers against incorporating evidence-based practice in physical therapy in Colombia: current state and factors associated

**DOI:** 10.1186/s12909-015-0502-3

**Published:** 2015-12-12

**Authors:** Robinson Ramírez-Vélez, M. Caridad Bagur-Calafat, Jorge Enrique Correa-Bautista, Montserrat Girabent-Farrés

**Affiliations:** 1 Centro de Estudios en Medición de la Actividad Física (CEMA), Escuela de Medicina y Ciencias de la Salud, Universidad del Rosario, Bogotá, D.C Colombia; 2 Departamento de Fisioterapia, Universitat Internacional de Catalunya, Barcelona, España

**Keywords:** Evidence-based practice, Barriers, Physical therapy, Survey

## Abstract

**Background:**

Evidence-based practice (EBP) has been widely implemented in differing areas of physiotherapy. Nevertheless, few studies have investigated EBP-related barriers amongst Latin-American physical therapists working in primary care. The primary objective of this study was to describe the current state concerning perceived barriers engagement in EBP among physical therapy in Colombia. A secondary objective was to identify factors associated with barriers to implementation EBP.

**Methods:**

A cross-sectional online survey was conducted. The study involved physical therapists working in public and private hospital who were contacted through professional networks (Email, Facebook_®_, ResearchGate_®_ and Linked-In_®_) and invited to participate. Multiple logistic regression (MLR) and multiple correspondence analysis (MCA) were used for examining factors associated with perceived barriers to including EBP in their work.

**Results:**

The final sample size was 1064 (77.2 % female). Forty-one percent of the respondents indicated that a “lack of research skills” was the most important barrier to evidence being used in practice. MLR analysis suggested that several variables were associated with perceived barriers to including EBP: i.e. hours of work per week, current main role in therapy center and undergraduate degree. The MCA model established two groups of similarities regarding the different barriers; the "lack of understanding of statistical analysis", "insufficient time" and "understanding of the English in which articles are written" barriers were weighted more heavily regarding in the first group (the second factor on MCA) and the rest barriers on the second group (first factor on the MCA).

**Conclusions:**

Although most physiotherapists had a positive opinion regarding EBP, they considered that they needed to improve their knowledge, skills and attitudes towards EBP. Initiatives to advance EBP in Colombia with no academic or research tradition should primarily target practitioner-level factors.

## Background

Evidence-based practice (EBP) is defined as integrating clinical expertise, patient values and the best available research evidence concerning decision-making for patient care [1, 2]. Regarding physiotherapy (PT), there is growing acceptance of an EBP-based approach which is referred to hereinafter as evidence-based physical therapy (EBPT) [3]. Along with PTs’ positive response to EBPT, a series of initiatives has been proposed which have been designed to generate, evaluate and disseminate research and put such results into practice [4, 5]. However, as the amount of PT research has increased dramatically during recent decades this has made it difficult for many PTs to keep up with advances in research and put findings into practice [6].

Most research has been focused on the use of evidence including a critical review of studies and scientific literature in clinical practice [7]. Nevertheless, in countries such as Australia [8], Spain [9], the United States [10], Colombia [11, 12] and the United Kingdom [13] the use of EBP, its teaching and evaluation in clinical practice has now been included in educational curricula.

The pertinent literature already contains questionnaires on EBP engagement [7–13]; to account for differences between Colombia [11, 12, 14] and the countries in which such questionnaires have been developed existing questionnaires must be culturally adapted to this setting, thereby ensuring their relevance for new contexts. Despite the clear benefits of EBP, its uptake within physiotherapy (and other healthcare domains) has been patchy and inconsistent in quality. Furthermore, practice may even be marked by variability within the profession (i.e. PT) which would depend upon existing laws, policy and conventions and hence can affect research evidence uptake [9]. Chapeton et al. [14], have described variations regarding the extent to which independent clinical decisions can be made by hospital-based Colombian PTs, therefore suggesting variations in how such decisions are made in ‘real world’ practice.

If we want the use of EBP to become a reality, especially in professions such as PT having a smaller research base and fewer resources compared to other professions such as medicine and nursing [15], then the numerous perceived barriers to using research in clinical settings must be examined, despite the lack of studies on this topic as revealed by a systematic review of EBP in physiotherapy by Fernández-Domínguez et al. [7].

Barriers are typically context-dependent; implementation strategies should thus be tailored according to their context and specific barriers must be identified [16, 17]. The most frequently reported barrier concerns limited time, thereby constraining the identification and interpretation of research evidence; being able to apply research findings to clinical practice has also been reported [9, 18]. Tilson et al. [19], have recently identified other common barriers, including an inability to determine the legitimacy of research findings, insufficient research literature on specific patients’ problems, deficient information regarding retrieval skills and an inability to incorporate patient preferences into decision-making, such results coinciding with those reported by Jette et al. [10].

A questionnaire developed by Jette et al. [10], regarding physiotherapy, was aimed at describing EBPT-related attitudes, beliefs, knowledge and behaviour; this involved extensive examination of existing guidelines [6–10, 20].

Jette et al.’s questionnaire [10] has been validated in Colombia for Colombian PTs by Flórez-López et al. [20]; these authors have shown high overall internal consistency (Cronbach values > 0.915), acceptable reproducibility (Test-retest 0.403 to 0.720) and appropriate construct validity (Kaiser-Meyer-Olkin >0.933; *X*^2^ = 20,366.877 and exploratory factorial analysis identified two factors accounting for 37.953 % of variance).

Investigators have used this instrument since, leading to a corpus of research findings being compiled documenting barriers to research use regarding continents, time and study setting [10, 21–23]. However, there is a paucity of well-developed, readily available, comprehensive measuring instruments serving as a resource for identifying EBP use or the barriers against and/or facilitators for using EBPT in the field of physiotherapy, particularly in healthcare settings. As reported by Fernández-Domínguez et al. [7], this has led to mistakes being made or existing deficiencies regarding the construction and/or validation of the original tool being ‘inherited’ in the new instruments so produced and, in many cases, no measures have been established which would enable their rectification.

Increasing numbers of patients in Colombia are being treated at primary care facilities, as in many other Latin-American countries; this includes PT outpatient clinics to which patients have direct access [11–15]. Providing PTs with the opportunity to conduct research could be a way of building evidence which can be used to keep the physiotherapy profession informed, change attitudes towards research and develop research skills. However, physiotherapists’ perceptions of barriers against their participation in research in their own filed are currently unknown. Knowledge concerning determinants of EBPT in Colombian primary care PT is limited [17, 23, 24]; little research has been published about PTs’ EBPT-related skills and knowledge or their perceived barriers to using EBP [11, 12, 17, 23, 24]. There has only been one quantitative study to date involving around 250 PTs in Colombia [12].

Our group has detailed Colombian PTs’ attitudes and beliefs regarding EBP in a previous article [12, 21]. In the past, the efforts to implement evidence-based practice in PTs have focused on describing potential barriers to the utilisation of research evidence among general therapies staff and reducing such barriers [6]. The primary objective of this study was to describe the current state concerning perceived barriers engagement in EBP among Colombian PTs. A secondary objective was to identify factors associated with barriers to implementation EBP.

## Methods

### Study design

This research involved using a cross-sectional survey whose descriptive design involved a web-based questionnaire; the questionnaire was based on previously published literature [6–10, 22]. The methods and results have been reported according to the Checklist for Reporting Results of Internet E-Surveys [25].

### Participants and sampling frame

Practising PTs in Colombia were targeted. Individuals were eligible if they met the following inclusion criteria: holding a diploma *or* BSc in physical therapy and currently providing physical therapy for patients which would account for some proportion of their time working in public and/or private hospitals. The Colombian Physical Therapy Association’s (in Spanish ASCOFI) membership list provided the sampling frame for this study (personal communication). Since not all Colombian PTs are members of the association, our sample for recruitment represents a convenience sample. The association has 500 members, and ≈ 12.000 no members of whom those were contacted via email and professional networks (Facebook_®_, ResearchGate_®_ and Linked-in_®_). Participants responded online and the survey software (SurveyMonkey_®_) added their responses to a results’ database in January 2012 and February 2013. The questionnaire consisted of 16 pages (screens) with 1–7 questions displayed per page. Participants were able to review or change responses using a back button. This led to 1250 visits to the web-site, 1064 of which were valid (response rate: 85 %).

### Questionnaire

The authors of the present study used a questionnaire designed in the United States by Jette et al. [10], which was validated and its reliability confirmed, as reported in a previous paper [20]. Ten multiple-choice questions concerning perceived barriers to EBP were classified into the following domains, based on previous research [10, 20]: 1- lack of research skills, 2- lack of understanding of statistical analysis, 3- inability to apply research findings to individual patients having unique characteristics, 4- insufficient time, 5- understanding of the English in which articles are written, 6- lack of information resources, 7- lack of collective support among my colleagues in my facility, 8- lack of interest, 9- poor ability to critically appraise the literature and 10- lack of generalisability of the literature findings to my patient population. For items on resources at work, response categories were ‘yes’, ‘no’ and ‘do not know’. All the barriers mentioned had been selected from a checklist and ranked on the basis of their importance, similar to existing questionnaires [10, 20]. The following demographic data was collected: gender, age, highest degree earned, current main role in therapy centre, hours of work per week, patients seen per day, type of facility and type of condition and age of most patients (Table 1). The time needed to complete the questionnaire was 15–20 min.

**Table 1:** Participant and practice characteristics

Characteristics	n (%)
Sex	
Male	243 (22.8)
Female	821 (77.2)
Age (y)	
20–29	845 (79.4)
30–39	155 (14.6)
40–49	58 (5.5)
50 +	6 (0.6)
Highest degree	
Undergraduate (Professional/graduate)	933 (87.7)
Specialized	116 (10.9)
Master’s	14 1.3()
Doctorate	1 (0.1)
Current main role in therapy centre	
Patient care	894 (84.0)
Clinical research	170 (16.0)
Hours of work per week	
< 20	114 (10.7)
20–30	256 (24.1)
31–40	349 (32.8)
> 40	345 (32.4)
Patients per day	
< 5	159 (14.9)
5–10	327 (30.7)
11–15	284 (26.7)
> 15	294 (27.6)
Type of facility	
Acute care hospital	175 (16.4)
Acute rehabilitation	55 (5.2)
Subacute rehabilitation	34 (3.2)
Skilled nursing facility	91 (8.6)
Private outpatient clinic	412 (38.7)
Facility-based outpatient clinic	124 (11.7)
Home care	36 (3.4)
School system	7 (0.7)
University	82 (7.7)
Other	48 (4.5)
Type of condition and age of the majority of patients	
Orthopedic	186 (17.5)
Neurological	64 (6.0)
Cardiovascular	630 (59.2)
Pediatric (<18)	62 (5.8)
Adult (19–64)	18 (1.7)
Geriatric (65+)	28 (2.6)
Sport	19 (1.8)
Other	18 (1.7)
No patient care	39 (3.7)

### Survey methodology

The SurveyMonkey_®_ online tool was used for creating the online questionnaire, as the link associated with the survey questionnaire and for collecting, storing and exporting the data (https://www.surveymonkey.com/s/JHNV89J). The questionnaire was not password-protected. The IP addresses of respondents were not recorded or stored and thus participants' responses remained anonymous. Prior to sending out the link, the usability of the link and the questionnaire were tested. The first step was to email a pre-notice to the target population announcing the chance to participate in answering the questionnaire in January 2012. Information regarding data handling (anonymity), informed consent, a description of the questionnaire (including the number of questions and expected duration), information about the research team and a link to the questionnaire was sent out later, via email and professional networks, along with an explanation of the study purpose. Multiple entries having the same IP address were not stored and/or limited because the idea was to enable several therapists using the same workplace computer to participate in this study, recognising that this might lead to multiple entries from the same participant.

### Ethical approval

This project was approved by the Universidad Manuela Beltran’s School of Health Ethics Committee for Research (N° 1008-2012-014) following ethical standards recognised by the Declaration of Helsinki and Colombian legislation governing research involving human beings (Colombian Ministry of Health resolution 008430/1993). A waiver of informed consent was granted as the participants were aware that by completing the questionnaire they were giving their informed consent. Participation in the study was totally voluntary and they had the option of declining to answer specific questions or to leave the entire questionnaire blank. All data were kept confidential and data protection was observed at all stages of the study.

### Data analysis

Descriptive statistics were used for analysing frequencies and distributions. All data was de-identified to maintain confidentiality. The categories were collapsed prior to examining associations between variables. Likewise, Jette et al.’s [10] “do not know” category was combined with the “no” category, based on our belief that lack of knowledge (for example, about whether a facility had access to the Internet) was as unhelpful to a respondent as not having access. Binary logistic regression analyses were conducted to examine the association of participant and practice characteristics with the barriers outcome measures. We used a forward selection process to derive models, requiring *p* < 0.05 to enter and *p* < 0.10 to delete. Odds ratios and their 95 % confidence intervals were recorded for each level of the independent variables that were significant. We chose one level of each variable as a reference group to allow the most salient interpretation of results. We used multiple correspondence analyses (MCA) to explore similarities and distances between barriers. It should be noted however that MCA is an exploratory technique, based on a philosophical orientation that emphasizes the development of models that fit the data, rather than the rejection of hypotheses based on the lack of fit (Benzecri's 'second principle'). Therefore, statistical significance tests are not customarily applied to the results of a correspondence analysis, and are not needed for the clustering of factors produced in a correspondence analysis. IBM SPSS_®_ statistics software (version 22.0, IBM Corp, Armonk, New York, USA) was used for statistical analysis.

## Results

### The participants’ characteristics

The final sample size was 1064 yielding a response rate of 85 % and a completion rate of 0.9 (number of PTs submitting the last questionnaire page divided by the number of PTs agreeing to participate). Table 1 summarises the participants’ characteristics; 79.4 % of the sample ranged from 20 to 29 years-old. PTs’ clinical practice was characterised by lasting more than 30 h a week (65.2 %). Time spent at work during an average month was divided between patient care (mean 84.0 %) and research-related activities (16.0 %). The number of patients seen by a therapist was between 5 and 10 patients (30.7 %) per day or more than 11 per day (54.3 %). Most patients were receiving cardiovascular/critical care (59.2 %) or care for respiratory conditions.

### Barriers to using evidence-based practice

The participants selected and ranked clinician-related barriers to using EBP (Table 2); 56 % of the respondents indicated “lack of research skills” as being the most important barrier to using EBP. The respondents rated “lack of understanding of statistical analysis” (52.8 %) and “an inability to apply findings to individual patients having unique characteristics” (46.9 %) as significant barriers. “Lack of generalisability of the literature findings to my patient population” was chosen as a significant barrier by 10 % of the respondents.

**Table 2:** Barriers to research evidence use by ranked importance

Barriers to EBPT	n (%)
Q-1: Lack of research skills	592 (56.0 %)
Q-2: Lack of understanding of statistical analysis	562 (52.8 %)
Q-3: Inability to apply research findings to individual patients with unique characteristics	499 (46.9 %)
Q-4: Insufficient time	463 (43.5 %)
Q-5: Understanding of the English in which articles are written	352 (33.1 %)
Q-6: Lack of information resources	218 (20.5 %)
Q-7: Lack of collective support among my colleagues in my facility	160 (15.0 %)
Q-8: Lack of interest	116 (10.9 %)
Q-9: Poor ability to critically appraise the literature	114 (10.7 %)
Q-10: Lack of generalizability of the literature findings to my patient population	107 (10.1 %)

### Factors associated with barriers against using evidence-based practice in daily practice

Table 3 shows the results for a binary logistic regression analyses. The physiotherapists that answered to the survey “insufficient time” OR 1.48 (95 % CI 1.03 to 2.16), "lack of information resources" OR 2.07 (95 % CI 1.15 to 3.71) and "understanding of the English in which articles are written" OR 2.88 (95 % CI 1.17 to 4.10) were associated as a barrier to implement EBP in current main role in a therapy centre factor within the patient care level. Undergraduate (professional/graduate) were associated as a barrier “lack of research skills” OR 1.57 (95 % CI 1.04 to 3.25) and “lack of understanding of statistical analysis” OR 1.90 (95 % CI 1.22 to 2.96). Lasted, “understanding of the English in which articles are written” was associated as a barrier when a PTs attends more than 15 patient’s per day OR 1.95 (95 % CI 1.99 to 3.19).

**Table 3:** Factors associated with barriers to implementation EBP

Barriers	Factor	Level	Odds Ratio (95 % CI)^a^	Model P	Model R^2,b,c^
Q-4: Insufficient time	Age (y)	20–29	1.98 (0.30–0.92)	<0.001	0.302
		30–39	Reference		
	Highest degree (Specialized)	No	0.56 (0.36–1.02)		
		Yes	Reference		
	Current main role in therapy centre	Patient care	1.48 (1.03–2.16)		
		Clinical research	Reference		
Q-6: Lack of information resources	Hours of work per week	<20	Reference	<0.001	0.211
		20–30	0.67 (0.38–1.19)		
		31–40	1.10 (0.70–1.72)		
		>40	2.07 (1.09–3.90)		
	Current main role in therapy centre	Patient care	2.07 (1.15–3.71)		
		Clinical research	Reference		
Q-1: Lack of research skills	Highest degree	No	Reference	<0.001	0.403
	Undergraduate (Professional/graduate)	Yes	1.57 (1.04–3.25)		
	Current main role in therapy centre	Patient care	1.24 (0.78–1.97)		
		Clinical research	Reference		
Q-9: Poor ability to critically appraise the literature	Highest degree	No	0.48 (0.27–0.85)	<0.001	0.204
	Undergraduate (Professional/graduate)	Yes	Reference		
Q-3: Inability to apply research findings to individual patients with unique characteristics	Hours of work per week	<20	1.49 (0.63–3.54)	<0.001	0.167
		20–30	1.40 (0.69–2.84)		
		31–40	2.57 (1.44–4.59)		
		>40	Reference		
Q-10: Lack of generalizability of the literature findings to my patient population	Current main role in therapy centre	Patient care	0.69 (0.37–1.30)	<0.001	0.201
		Clinical research	Reference		
Q-2: Lack of understanding of statistical analysis	Age (y)	20–29	0.57 (0.33–0.10)	<0.001	0.507
		30–39	Reference		
	Highest degree	No	Reference		
	Undergraduate (Professional/graduate)	Yes	1.90 (1.22–2.96)		
	Hours of work per week	<20	0.41 (0.22–0.77)		
		20–30	0.97 (0.59–1.59)		
		31–40	0.68 (0.45–1.02)		
		>40	Reference		
	Current main role in therapy centre	Patient care	0.67 (0.42–1.08)		
		Clinical research	Reference		
Q-7: Lack of collective support among my colleagues in my facility	Current main role in therapy centre	Patient care	0.68 (0.43–1.08)		
		Clinical research	Reference		
Q-5: Understanding of the English in which articles are written	Sex	Male	0.43 (0.27–1.68)	<0.001	0.235
		Female	Reference		
	Highest degree	Yes	0.61 (0.40–0.92)		
	Undergraduate (Professional/graduate)				
		No	Reference		
	Hours of work per week	<20	0.41 (0.22–0.76)		
		20–30	0.97 (0.59–1.59)		
		31–40	0.68 (0.45–1.02)		
		>40	Reference		
	Patients per day	<5	Reference		
		5–10	1.12 (0.62–2.03)		
		11–15	1.48 (0.91–2.41)		
		>15	1.95 (1.19–3.19)		
	Current main role in therapy centre	Patient care	2.88 (1.17–4.10)		
		Clinical research	Reference		

^a^95 % CI confidence interval

^b^In binary logistic regression, one level of the independent variable serves as a reference against which the odds of the other levels occurring are determined

^c^Nagelkerke *R*^2^

### Multiple correspondence analysis

**Fig. 1:**
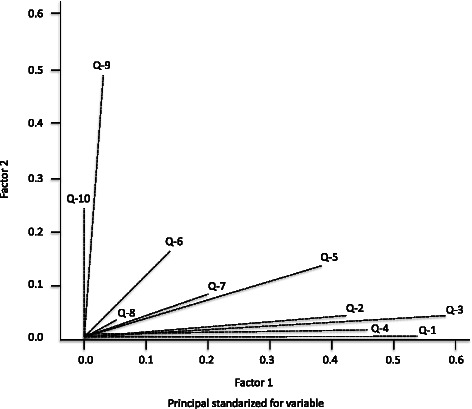
Reduction of barriers to EBP. This meant that each factors was a linear combination of the 10 barriers, each barrier’s corresponding weighting (0 to 1) regarding each factor being the coefficient of such linear combination. Each of the 10 barriers could then be represented on a 2-dimensional figure (map), such representation leading to the detection of clusters/groupings between different barriers, based on the distances and/or similarities between them, derived from similarities in respondents’ answers. Q-1: Lack of research skills, Q-2: Lack of understanding of statistical analysis, Q-3: Inability to apply research findings to individual patients with unique characteristics, Q-4: Insufficient time, Q-5: Understanding of the English in which articles are written, Q-6: Lack of information resources, Q-7: Lack of collective support among my colleagues in my facility, Q-8: Lack of interest, Q-9: Poor ability to critically appraise the literature, Q-10: Lack of generalizability of the literature findings to my patient population

MCA allowed the 10 barriers to be summarised in the same amount of possible factors (dimensions) with the least loss of information (i.e. 2 factors), considering the maximum variability explained. The MCA model explained 43.6 % variability between the two factors (31.1 % regarding the first factor and 12.5 % for the second one), Fig. 1.

**Fig. 2:**
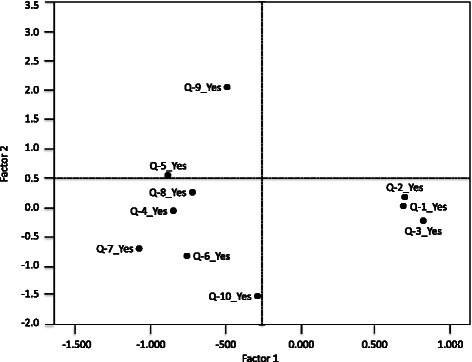
Multiple correspondence analyses for representation of barriers to EBP. Q-1: Lack of research skills, Q-2: Lack of understanding of statistical analysis, Q-3: Inability to apply research findings to individual patients with unique characteristics, Q-4: Insufficient time, Q-5: Understanding of the English in which articles are written, Q-6: Lack of information resources, Q-7: Lack of collective support among my colleagues in my facility, Q-8: Lack of interest, Q-9: Poor ability to critically appraise the literature, Q-10: Lack of generalizability of the literature findings to my patient population

Figure 2 shows that Q9 (poor ability to critically appraise the literature) and Q10 (lack of generalisability of the literature findings to my patient population) were the barriers having the greatest weighting in the second of the factors. Q1 (lack of research skills), Q2 (lack of understanding of statistical analysis), Q3 (inability to apply research findings to individual patients with unique characteristics) and Q4 (insufficient time) were the barriers having the greatest weighting for the first of the factors. This indicated that respondents’ answers to Q9 tended to be the same as to Q10. The same thing happened with Q1, Q2, Q3 and Q4, whilst the rest of the barriers concerned aspects which would have had quite similar weighting regarding both factors or, rather, without these being determinants for either factor. Respondents who considered that Q4 (insufficient time) was a barrier for EBP usually thought the same regarding Q5 (understanding of the English in which articles are written), Q6 (lack of information resources), Q7 (lack of collective support among my colleagues in my facility) and Q8 (lack of interest). Figure 2 also shows how these barriers were placed in a group having small distances between them. Likewise, respondents giving Q1 (lack of research skills) as a barrier can be detected in the figure at a very short distance from those also considering that Q2 (lack of understanding of statistical analysis) was a barrier and also at a short distance from those considering Q3 (inability to apply research findings to individual patients with unique characteristics) to be a barrier. These two groups are clearly differentiated on the map given in Fig. 2, indicating that they had different views of what for them were the main barriers against using PBE. However, barriers Q9 (poor ability to critically appraise the literature) and Q10 (lack of generalisability of the literature findings to my patient population) at the other extreme of the map were the barriers against EBP which were not usually detected as such by most respondents.

## Discussion

Quantitative and qualitative surveys [6–10, 21–26] and several countries’ health authorities [23] have promoted making EBP the standard approach for providing health services. However decision-making must also take into account the wishes, expectations, and values of the patient as well as the therapist's experience and knowledge [1–5, 24]. Barriers limiting EBP use in a particular profession must thus be identified so that strategies might be proposed to facilitate using EBP from training stages onwards [26].

Although reliable national data regarding the profile of Colombian PTs is currently lacking, it can be considered that our sample was fairly representative of most practitioners as has been previously described among physical therapists [9–22]. This study has provided a clear rationale for the further development of methods for identifying barriers among Colombian PTs. However, the actual contribution of barrier analysis for developing implementation strategies is still considered a black box.

Quantitative assessment of barriers via surveys and qualitative methods via in-depth interviews are thus often combined and allow for in-depth analysis [9, 25]. A sample containing a very high prevalence of being young, having few years of practice and just undergraduate-level education by contrast with samples from developed countries [3–10], was consistent with the PT turnover rate observed in Colombia, as many PTs seek employment overseas or move into financially-rewarding fields. For example, Colombian PTs’ job turnover rates are often much higher and there are fewer long-lasting jobs. Future multicentre studies in Colombia should be aimed at confirming a link between high turnover pressure, being younger, fewer years in practice and just having had undergraduate-level education.

A surprising finding was that 87.7 % of the PTs in this study had received no formal postgraduate education, such percentage being similar to that reported by Gorgon et al. [26], (90 %) in the Philippines and higher than that reported by Jette et al., in the USA (40–67 %) [10]. In Latin-American, these discrepancies may be explained by differences in level of academic preparation and access to educational initiatives (e.g. not all professionals have access to a professional development programme) [11, 12, 21, 22]. Lastly, although Colombian PTs may believe EBP is important, they may not feel it istheir responsibility to undertake all its steps.

Many researchers have discussed barriers against continuing professional development, i.e. physical barriers, attitude-based barriers and structural barriers, or similar categories of situational, institutional and dispositional barriers. Postgraduate education is thus important for Colombian PTs, especially because of rapid innovation and advances in the discipline, as well as on-going progress in science concerning rehabilitation and specialisation [11, 12, 14]. Structural barriers to postgraduate education and further professional development can be regarded as being synonymous with institutional or organisational barriers, i.e. practice, procedures and policies imposing limits on opportunities for potential adult learners to participate. Research among doctors has identified only a small number of professionals having poor motivation regarding their own further education [14]. This is a common scenario in Colombia and explains the high score for organisational barriers noted in this study.

As in other work, the main obstacle to using EBP lay in “a lack of research skills” [6–10]. In Colombia, the physiotherapists recognized that changing clinical practice and research skills are a process that takes time [11, 12, 14]. Various solutions have been proposed in the literature, but there appears to be consensus that time must be set aside to provide a formal, scheduled opportunity to meet and discuss relevant research-related matters and that meetings should focus on reflection on research findings and clinical guidelines rather than discussions based on experiential or anecdotal knowledge not linked to research [6, 24].

Barriers which have been reported by researchers in developed countries were also identified [3, 5, 6, 10–12, 14], such as “understanding the English in which articles are written”, “an inability to apply research findings to individual patients having unique characteristics”, “insufficient time” and “a lack of requisite skills” (e.g. searching for, appraising, interpreting statistics, interpreting research results and applying research findings to patients) and “a lack of collective support among the colleagues in my facility”. Changing the hospital team’s attitude towards clinical practice was considered the least difficult barrier. The profession of physiotherapy in Colombia has traditionally been based on the theory of guided practice by contrast with medicine which is dominated by empirical models regarding evidence, especially that obtained from randomised controlled trials [11–13]. However, Bernhardsson et al. [24] have argued that more research is needed into various aspects of the “lack of time” concept before it is possible to reduce the impact of this factor on implementation of EBP. Limited time is certainly not unique to physiotherapy or healthcare in general, as there is a difficult trade-off between short-term production requirements and longer-term ambitions for learning and development in many work contexts.

On the other hand, MCA for representing barriers against EBPT has shown that "a lack of understanding statistical analysis", "insufficient time", "understanding of the English in which articles are written", “lack of interest”, “poor ability for critically appraising the literature” and “a lack of generalisability of the literature findings to my patient population” were significant barriers (Group 2, Fig. 2). Most barriers cited by PTs (Group 2) were considered to be internal barriers beyond their control, although the lack of self-criticism expressed was remarkable, as has been pointed out by Grimmer-Somers concerning Australian physiotherapists [27]. However, as the demands on healthcare professionals seem unlikely to be alleviated in the future, this review focuses on the other consistent but modifiable barriers identified in the research, as potential areas for intervention. If such modifiable barriers are addressed, this may have a positive effect on time/workload pressures, as well as enhancing the provision of appropriate and effective care [27–29].

These barriers reflected the mental time and energy required for using research and the culture of busyness rather than the actual amount of time required. Colombian PTs and the organisation itself maintain a culture in which busyness is valued and rewarded. This may be related to personal characteristics such as experience and confidence and how tasks can be solved in a complex organisation [24]. The lack of confidence regarding critical appraisal skills in half of the participants paralleling that of previous surveys [3–14] appeared to be closely related to the fact that half of them had not received critical appraisal training. Critical appraisal, while highly important in research evidence uptake, requires complex skills which take time to develop and self-efficacy would be likely to become increased following upskilling in critical appraisal [26].

Similarly, “an inability to apply research findings to individual patients having unique characteristics”, “a lack of information resources” and “a lack of collective support among colleagues in my facility” were statistically significant and grouped with barriers against EBPT use (Group 3, Fig. 1) in MCA. A plausible interpretation would concern some participants having reported confidence regarding their use of generic electronic databases but who were not sufficiently aware of where and how to locate EBPT-related information [26]. Research is not used in isolation but is influenced by factors at individual level, collaboration between multidisciplinary groups, management and organisational structure. Rubio-Valera et al. [25], in a recently updated systematic review, found an association between the use of research and individual factors such as attitudes towards EBPT, having completed a course in evidence-based practice, undergraduate education, working in a therapy centre and job satisfaction.

A surprising result was that the barrier against using EBPT as a resource in practice decreased with PTs having aged 20–29 years and the number of work hours per week. This finding is similar to reported by Durán-Palomino et al. [30] and Ramírez-Vélez et al. [22, 31] in Colombian physiotherapists (i.e. near 85 % PTs not attended external meetings and/or took part in networks and regional/national conferences at which research in the last year in the moment to enrolled study, age mean 28,7 ± 4,2 years old). They considered this exchange of knowledge and experience with other physiotherapists to be very important for their competence development and commitment to using research in daily clinical practice. This contrasted with work by Jette et al. [10], who reported that the attitude of younger practitioners with less work experience is more positive compared to older participants with longer years of experience.

Given that PTs reported that research literature acquired status and was put into practice when local consensus reasoned that it fit in with their on-going work, support for EBPT from colleagues and others within a work facility are quite important [10]. Chapeton et al. [14], have shown that PTs resist using such activities, citing barriers concerned with clinical practice such as excessive workload, lack of skills and knowledge, professional-patient relationship-related problems and a lack of confidence regarding the effectiveness of such interventions. Most of these barriers can be overtaken with training as well as by promoting workplace changes with regard to a better use of EBP.

Only 5 % of a sample of Colombian physiotherapists (*in Spanish* ASCOFI), were found to be members of the major professional association, a theme replicated in other samples (*data no shown*). Professional education seminars on EBPT are offered only sporadically in Colombia by ASCOFI and some higher education institutions; PTs can renew their professional license without engaging in continuing professional education. Some research [25, 29] revealed that although many felt membership was important and provided their profession with a voice, a significant minority felt disillusioned with the provision (particularly regarding prohibitive costs), leading to ambivalence towards membership.

This contrasts with the scenario in many developed countries where continuing professional education programmes are more readily available and well-placed and pre-professional educational content promoting the use of research evidence in daily practice has recently been included in curricula [28]. It appears that a logical next step would be to move towards developing educational programmes which can help Colombian PTs to improve their knowledge and skills.

Some PTs have stated that clinical practice guidelines represent *“cookbook medicine”* and do not allow for clinical judgment as their reason for not following or using them [10]. Another review [29] has showed that physiotherapists tend to present favourable opinions toward EBP and the mainly barriers faced by them are usually related with lack of time and skills, and also misperceptions of EBP. Some factors which have been traditionally identified in the literature as barriers to EBP were cited by a minority of respondents, such as a lack of information resources [32]. Although use of research evidence in EBP has been previously described for developed countries [3–6], little is known about it in developing countries such as those in Latin-America [22, 30, 31].

This study involved several potential limitations. First, the instrument and variables used in this study were selected following a review of the pertinent literature. However, as with any other study, just a finite number of questions were included. Another limitation concerned the participants’ personal characteristics not being taken into account, as work by several authors has shown that they significantly modulate an inclination to use EBP. The study’s exploratory rather than in-depth nature, the non-random sample selected and the use of self-reported data represent other limitations, as did the potential bias introduced by the sampling frame. The study’s cross-sectional nature did not allow for a deep exploration of the extent to which the participants had been educated/trained regarding EBP. The sample was drawn from Colombian PTs working in predominantly metropolitan areas; it was therefore unclear whether and to what extent the results reflected research evidence uptake in rural areas. Given the current professional emphasis on EBP, respondents might have addressed items in a socially acceptable manner; they might have reported more positive attitudes and beliefs and higher levels of knowledge than they actually had. Gender differences were not analysed. The variability found in this study did not necessarily imply that Colombian PTs were improperly using EBP in clinical practice; this possibility should be determined by studies having mixed design and approaches. Such statements can only be made if there is agreement on use, ideally regarding how to use web-based clinical practice guidelines. Decision-making requires knowing about and managing the elements and stages shaping EBP, as well as the barriers against it and strategies for using it; however, such limitations did not compromise the results when validating them [33, 34].

Making clinical decisions involves a personal dimension related to the professionals taking them; however, most professionals work within a health service regulatory and organisational framework [10–15, 34]. Clinical administrators should increase the availability of computer access to research databases and provide time for clinicians to search for and read the pertinent literature and/or communicate research findings among their colleagues.

A major strength of this study lies in it being the first work to provide information concerning perceived barriers to incorporating EBP by a group of PTs working in Colombia. This study would suggest that future research should include studies aimed at examining the actual processes through which evidence is gathered, synthesised and used by PTs in various settings, as well as related demographic factors.

## Conclusion

Our results show that strategies should be developed to support PTs regarding changes in their behaviour towards a more comprehensive use of research in clinical work. The results are generalisable to other countries having no research or academic tradition. Schreiber and Stern have pointed out the important role of professional organisations in EBP becoming implemented in daily practice [33]. Associations should aim at changing attitudes, offer PTs CE regarding specific topics and provide EBP resources, such as access to databases or links to guidelines. This study has thus confirmed that most Colombian PTs in the sample had a positive opinion about EBP and that they considered that they needed to improve their knowledge, skills and attitude towards it.
